# Dynamic osteosynthesis by modified Kuntscher nail for the treatment of tibial diaphyseal fractures

**DOI:** 10.4103/0019-5413.48824

**Published:** 2009

**Authors:** Wasudeo M Gadegone, Yogesh S Salphale

**Affiliations:** Department of Orthopaedic and Traumatology, Chandrapur Multispeciality Hospital, Mul Road, Chandrapur 442401, India

**Keywords:** ‘D’ interlocking nail, dynamic osteosynthesis, intramedullary nail, modified Kuntscher nail, tibial diaphyseal fracture

## Abstract

**Background:**

We evaluated a series of diaphyseal fractures of the tibia using low-cost, Indian-made modified Kuntscher nail (Daga nail) with the provision of distal locking screw for the management of the tibial diaphyseal fractures.

**Materials and Methods:**

One hundred and fifty one consecutive patients with diaphyseal fractures of tibia with 151 fractures who were treated by Daga nail were enrolled. One of the patients who had died because of cancer, and the two patients who were lost to follow-up at 3 months were excluded from the study.Therefore data of 148 patients with one hundred and fortyeight fractures is described. One hundred twenty closed fractures, 20 open Grade I fractures, and eight open Grade II fractures as per Gustilo and Anderson classification were included in this study. One hundred fourteen men and 34 women, with a mean age of 38.4 years, were studied. The result were analysed for Surgical time, duration of hospitalisation, union time, union rate, complication rate, functional recovery and crutch walking time. The fractures were followed at least until the time of solid union.

**Results:**

The follow-up period averaged 15 months (range, 6–26 months). Union occurred in 140 cases (94.6%). The mean time to union was 13 weeks for closed fractures,17.8 weeks for Grade I open fractures, and 21.6 weeks for Grade II open fractures. Compartment syndrome occurred in two patients. Superficial infection occurred in five cases of Grade I and II compound fractures. Three closed fractures and one case of Grade I compound fracture required bone grafting for delayed union. Two cases of Grade II compound fracture with nonunion required revision surgery and bone grafting. Twelve cases resulted in acceptable malalignment due to operative technical error. In four cases, the distal screw breakage was seen, but none of these complications interfered with fracture healing. Recovery of joint motion was essentially normal in those patients without knee or ankle injury.

**Conclusion:**

Unreamed distally locked dynamic tibial nailing (modified Kuntscher nail/Daga nail) can produce excellent clinical results for diaphyseal tibial fractures. It has the advantages of technical simplicity, minimal cost, user-friendly instrumentation, and a short learning curve.

## INTRODUCTION

Many alternatives for treatment of fracture of the tibial diaphysis are available these days. Each treatment method, whether it is closed reduction and immobilization, functional cast brace,^1^ medullary nailing^2^ osteosynthesis with plates, or external fixation,^3^ has its advantages and disadvantages. The indications for their usages vary widely and also depend on the operating surgeon's preference.

The use of closed intramedullary nail after reaming of the tibia has become an acceptable standard worldwide.^2^ This method is technically demanding with sophisticated instrumentation set, costly implants, and steep learning curve, which are not economically feasible in the developing countries for a variety of reasons.

The successful use of Indian-made tibial nails, known as D interlocking nail (modified Kuntscher nail), in certain closed tibial fractures stimulated us to use the method of intramedullary nailing (Sapana Surgicals, Solapur,India). (The nail is made up of stainless steel 316/316 and has bending force of 52 kg/cm^2^).

We performed a retrospective review of our 6-year experience with 148 patients (148 tibial diaphyseal fractures) treated with unreamed, tight-fitting, modified Kuntscher nail for closed and Grade I and II open fractures as Gustilo and Anderson classification. The results were evaluated and compared with those of the existing standard for interlocked nailing systems.

The aim of this article is to evaluate a simple, user-friendly, Indian-made intramedullary nail for the management of the fractures of the tibial diaphysis performed with minimum instrumentation, respecting the biomechanical principles and achieving an eventually good functional outcome comparable to the existing standard surgical techniques.^2^,^4^,^5^

## MATERIALS AND METHODS

The method was used in 148 patients with 148 fractures at Chandrapur Multispeciality Hospital from July 2001 to July 2007. One hundred forty-eight consecutive patients with 120 closed fractures, 20 Grade I open fractures, and eight patients with Grade II open fractures were included in this study. One hundred fourteen men and 34 women, aged between 18 to 72 years (mean, 38.4 years), were studied. The tibial fractures were classified according to the location [Table 1], and the patterns of fractures were analyzed according to the Arbeitsgemeinschaft fur osteosynthesefragen–Orthopaedic Trauma Association (AO/OTA) classification.

**Table 1:** The site of fractures

Site of fracture	Patients N	Males N	Females N
Lower fourth	10	6	4
Upper third	8	6	2
Middle third	80	60	20
Lower third	38	33	5
Segmental	12	9	3
Total	148	114	34

In our study, the most common location of the fractures was the middle third of the tibial shaft. Type A fractures were 74, Type B were 46, and 28 fractures were of Type C. The middle part of the tibial shaft was the most often involved fracture site in all the categories.The causative fractures are summarized in Table 2.

**Table 2:** Associated fractures

Upper tibial plateau	3
Medial malleaolus	4
Calcaneum	5
Metatarsal	2
Ribs	3
Femur	9
Contralateral tibia	3
Forearm	7
Total	36

We used our method as primary treatment in 116 fractures within 72 h after presentation and in 32 patients within 10 days after initial injury. Sixteen patients were classified as multiple-trauma victims, who were operated after a delay of more than 72 h. A high-energy mechanism was responsible for the injury in 52% of the patients. A motor-vehicle accident was the cause in 101 patients, and 24 patients were pedestrians who were struck by an automobile. Falls and miscellaneous accidents caused injuries in the remaining 23 patients. Thirty-six patients had one or more additional fractures which are summarized in [Table 2]. Patients with head injury were excluded from the study. Eleven had an abdominal injury that required laparotomy.

The modified Kuntscher nail is similar to the standard Kuntscher nail with a few modifications, which is called D interlocking nail or Daga nail. The proximal end is strengthened and shaped like the letter “D” and has an eye at the proximal end to assess the rotation of the nail, for locking in a static mode as well as for its removal. The “D” is flattened anteriorly, and this prevents the anterior knee irritation and pain. It is shaped like a platform, which acts like beam over the two walls of nail. It takes the thrust of the hammer during the operation and weight-bearing. There is 8° Herzog bend, at its upper third situated at 5 cm from the proximal end. It has a slit of 3 mm at its posterior surface, the lower end goes on tapering and has a hole distally for interlocking screw fixation. The nail has a cross section of cloverleaf through out its length.

Of the One hundred and twenty closed fractures closed reduction, and nailing was carried out in 108 patients whereas twelve patients needed limited open reduction. In open fractures, wound debridement and nailing was carried out. Two patients needed soft tissue coverage. The nail size was determined by measuring the isthmus of the medullary canal with a ruler. The nail, 1.5 or 2 mm narrower than the isthmus, was chosen, as this allowed for a good fit while avoiding nail incarceration. The nail size of 9 mm was implanted in the majority of cases. Distally the nail was locked with a 4.5-mm locking bolt. Attempts to gain cortical contact between the fracture surfaces were made, and minimal shortening (< 1 cm) was accepted to achieve this goal.

The nailing was done under spinal anesthesia and image intensifier on a fracture table. The hip was flexed to 45° and knee up to 60–90° with a well-padded bolster kept away from the popliteal fossa to avoid pressure on the popliteal vessels. Manual traction is given by the assistant. Reduction was done and checked in anteroposterior and lateral views on the image intensifier.

A 2.5-cm incision was made in the midline at the upper end of the tibia just adjacent to the patellar tendon on the medial aspect. The patellar tendon was retracted laterally. The entry point of the nail was marked with a curved awl and the medullary cavity was reached gradually. The modified Kuntscher nail was gradually hammered reducing the fractures fragments and checking under the image intensifier till the tip anchors into the distal tibial metaphysis. The distal hole was then locked with a 4.5-mm cortical screw or a locking bolt using a free hand technique. The stability was assessed. In cases where we felt that distraction is likely or is seen on the image intensifier, we thumped the heel keeping the knee flexed and stabilized by the assistant to bring the fragments in good apposition.

**Figure 1:**
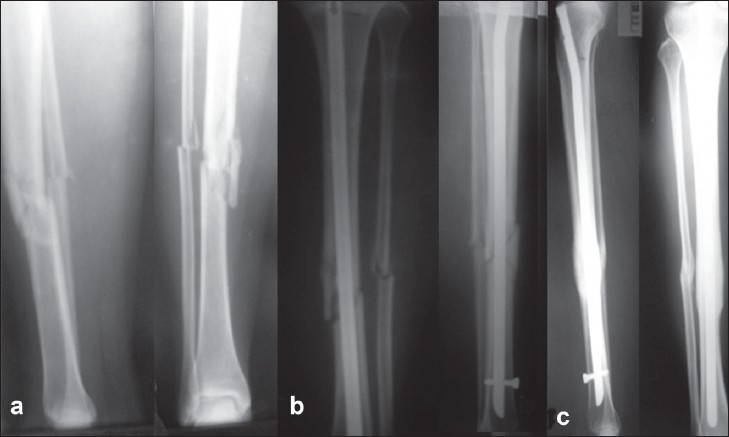
(a) X-ray of right leg lateral and anteroposterior views showing a fracture of both bones of leg, middle third with a butterfly fragment. (b) 6 weeks postoperative (anteroposterior and lateral) radiographs showing early healing, (c) radiograph showing good consolidation at 1 year follow-up.

In most cases the nail is selected that rests within 2 cm of the distal joint line in the antero posterior view and flush with the entry point in the proximal end. A well-padded dressing was given. Crepe bandage and a below knee slab was given till suture removal or till subsidence of the postoperative edema. The mean time for the surgery was 38 min (25–55 min) and the average hospitalization time in our cases was 10 days (7-20 days).We waited for the polytarumatized patients to be surgically fit to undergo the procedure. Modified Kuntscher nails/D nails of size 8 was used in 18 cases, size 9 in 90 cases, and size 10 in 34 and size 11 in six cases. The bolt used for the 9 mm nail was of 4.5 mm. whereas those used for the 8 mm nail is of 3.5 mm. All the nails were locked distally in an anteroposterior direction [Figure 1].

**Figure 2:**
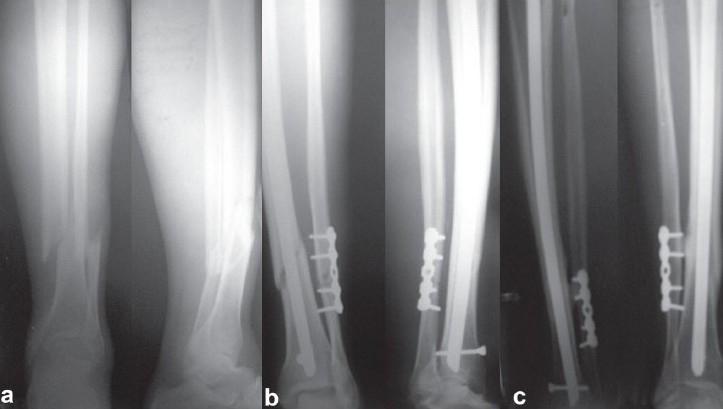
X-ray of left leg (anteroposterior and lateral views) showing a short oblique diaphyseal fracture of distal one fourth of tibia and fibula, which are inherently unstable. (b) Immediate postoperative (anteroposterior and lateral)radiographs showing good fracture reduction. (c) radiograph showing good consolidation at 9 month follow-up.

Plating of the fibula was carried in 14 cases where we felt that the distal tibial fragment was unstable [Figure 2a–b]. The unstable diaphyseal fractures and distal meta-diaphyseal fractures that were rotational unstable were stabilized with a poller screw.

Limited open reduction was required in 12 patients. In two cases, the nail was incarcerated with in the canal during our initial stages of the study. The nail was released by giving a small incision at the fracture site and performing a linear corticotomy. The nail was driven further and locked distally. The fracture healed without any complication. Two of the cases had compartment syndromes, which required immediate fasciotomy on the third day of the surgery.

The postoperative regime varied with the fracture pattern and associated soft tissue injury. Immediate weight-bearing was allowed for patients in whom the fracture was stable, with good rotational control for the nail with interlocking bolt. The patients were encouraged to perform quadriceps exercises and ankle and foot exercises as early as the pain became tolerable.

The fracture was assessed for union at the follow up examinations. Absence of pain at the fracture site and radiographic evidence of callus in anteroposterior and lateral views of two or more cortices was considered as healed.

## RESULTS

Follow-up evaluation was performed on 148 patients with 148 fractures. One patient died because of cancer, and two were lost to follow-up at 5 months and were excluded from the study. The fractures were followed at least until the time of solid union. The follow-up period averaged 15 months (range, 6–26 months).

Thirty-two patients were allowed weight-bearing after the removal of stitches at 2 weeks, and seventy-six were fully weight-bearing by 4 weeks after operation. A cast or a cast-brace was used in 40 patients indicated for type B (less stable), type C (most unstable) fractures, and then partial weight-bearing was begun when the fracture was stable to axial and rotational load at 8 weeks [Figure 1a–c]. A few patients (28 patients with type C) with the most unstable fractures used crutches for variable periods, and they were allowed progression to full weight-bearing over a 6–10 week period. The longest time to full weight-bearing was between 10 and 14 weeks (average, 12 weeks) postoperatively in 12 patients who had a segmental mid-diaphyseal fracture.The average time to full weight-bearing was 8.2 weeks. The average time to union of the closed fracture was 13 weeks (range, 6–40 weeks). The healing times for the primarily nailed compound Grade I and II averaged 17.8 and 21.6 weeks, respectively.

Three patients with closed fracture and one with Grade I compound fracture had delayed union of a primarily nailed fracture necessitating bone grafting after 12 weeks of clinicoradiological observation. They attained the full Function of ankle and knee. Two patients of Grade II compound fracture treated by nailing required bone grafting once the soft tissue healed.

We resorted to plating of the fibula in 14 cases, in which we thought that the stability of the distal tibial fragment was questionable. Fixation of the fibular fracture helped us to correct alignment of the tibia. Superficial infection occurred in five cases, three of which were on the nail entry portal whereas two were at the distal locking site. They were treated successfully with debridement of the wound and antibiotics. Distal screw breaking was seen in four cases probably because of the early weight-bearing, but the fracture healed uneventfully. Return of a functional range of motion in the knee and ankle, as measured at the last clinical visit at 6-monthly follow-up, was at least 80% of the range on the uninjured side. The loss of joint motion in the ankle of three patients was not related to the tibial fracture.

The fracture healed in varus angulation in seven cases. The angle ranged from 3–10°(mean, 5°), but that did not affect the appearance and the functional demands of the patients. In five cases, valgus amounting to 3–8° was not well compensated.

Eleven patients of comminuted fractures had a shortening of 1 cm. Fourteen patients had less than 1 cm of shortening ranging from 5–8 mm. The proximal migration of the nail was seen in seven cases, and the distal migration was seen in four cases with screw breakage. Eleven patients reported anterior knee pain. Three patients required early removal for pain and persistent irritation at the knee after union.

There was no nail breakage. In three cases, we could see the recurvatum deformity after the nail was bent, but it was under physiological limits and the patient had presented at the routine clinicoradiological review. Union occurred in these three cases without the need of resorting to secondary procedures. These were the patients of Type II compound injuries. (The results were graded according to the criteria laid down by Johner and Wruhs). Our results were excellent in 90 cases (60.8%), good in 50 cases (33.8%), and fair in eight cases (5.4%) [Table 3].

**Table 3:** The results in our study based on the criteria of Johner and Wruhs^13^

Criteria	Excellent	Good	Fair	Poor
Nonunion/infection	None	None	None	Yes
NV injury	None	Minimal	Moderate	Severe
Deformity (in degrees)				
Valgus/varus	None	2–5	6–10	>10
Pro/recurvatum (in degrees)	0–5	6–10	11–20	>20
Rotation	0–5	6–10	11–20	>20
Shortening	0–5 mm	6–10 mm	11–20 mm	>20 mm
Mobility				
Knee	Full	>80%	>75%	<75%
Ankle	>75%	>75%	>50%	<50%
Subtalar	None	>50%	<50%	
Pain	None	Occasional	Moderate	Severe
Gait	Normal	Normal	Mild limp	Significant
Activities: sternous	Possible	Limited	Sever restriction	Impossible
Results (n)	90	50	8	0

## DISCUSSION

It is still a matter of debate whether tibia diaphyseal fractures should be treated conservatively or not. Surgery is rarely required for the treatment of tibia fractures.^6^ Sarmiento *et al*.,^1^ in 1000 consecutive closed diaphyseal tibial fractures treated with functional bracing, concluded that the high union rate and low morbidity associated with functional bracing of closed tibial fractures should be considered rather than expensive surgery. External fixator is one of the treatment options for compound fractures of the tibia and has the advantage of being technically easy to perform, preventing soft tissue stripping, ease of removing hardware, and provision for caring the injured tissues.

The external fixator^2^ is associated with relatively high rate of complication such as pin tract and deep infections, delayed union, nonunion, and malunion, which ultimately limit the durability of the frame construct. More recently, a staged treatment in compound fractures, with initial application of spanning external fixators followed by definitive fixation at secondary phase, has been advocated.^7^ We have tried this method in four cases, and our experience is very limited.

Open reduction and internal fixation (ORIF) by plates and screws requires extensive soft tissue dissection, further devitalizing the soft tissue and bone. Longer mean time to union is expected in comminuted fractures treated by ORIF. Other complications such as wound breakdown, infection, loosening of the implants, and infected non-union have been reported.^8^

The locking compression plate (LCP) and the Less-Invasive Stabilization System (LISS) are the new implants with angular stability developed by the association of osteosynthesis/association for study of internal fixation. A precise preoperative planning and a good knowledge of biomechanics is essential for optimal results.^9^ Percutaneous plating featuring small incision, limited periosteal stripping, indirect reduction techniques is a biologically beneficial alternative to traditional plating techniques.^10^

Intramedullary nailing for the fixation of the diaphyseal fractures was introduced by G. Küntscher. Many centers across the world have been performing these surgeries with good results.^11^ Closed intramedullary nailing along with internal locking technique, which is now common in clinical use, is a desirable and biological osteosynthesis. There have been proponents and opponents claiming the superiority of the reamed nail over the unreamed technique. Brown *et al*.^12^ reported their results with reamed nailing on 128 acute displaced and Type I open fractures and had a union rate of 98% at an average of 17 weeks. The deep infection was 17.8%, and the malunion rate of 2.4%.

The excellent results of unreamed nailing with the solid tibial nail consequently led to its use as the primary method of treatment for tibial fractures associated with soft tissue injury.^13^ The AO universal tibial nail (UTN) inserted and interlocked without reaming allows the stabilization of complex fractures respecting the biomechanical aspects.^14^ The unreamed intramedullary nailing provides a safe and effective tibial stabilization in the acute multitrauma patients.^15^ Union time, hardware failure rate, and secondary procedures, however, appear to be increased compared with the previous studies of reamed nails. No reaming is done in any of our case, as we believe that there is damage to endosteum of medullary cavity, thinning of cortex, the possibility of adult respiratory distress syndrome or pulmonary embolism, and high infection rate.^16^ We agree with Tornetta *et al*.^7^ in that intramedullary nailing performed without reaming or traction is safe with respect to compartment syndrome. In our study, although we used the unreamed technique, we had acceptable rate of complications as mentioned in the literature for the reamed nailing.

Dynamization is essential for the union and preventing hardware breakage. The modified Kuntscher nail acts on the principle of three-point fixation in dynamic mode from the immediate postoperative period. Therefore, the rate of nonunion and screw breakage is very low in our study because of gradual weight-bearing as tolerated by the patient as compared with the other studies reported by various authors.^12^,^17^

Anterior knee pain has been reported in 40%.^18^ Although the etiology of anterior knee pain is multifactorial and warrants further study, it is observed that it is due to the irritation caused by proximal end of the intramedullary nail and dissection on the knee.^2^ Considering the minimal number of symptomatic patients with anterior knee pain in our study, we recommend a para patellar tendon incision for nail insertion, sufficiently burying the nail into the upper tibial metaphysis well below the patellar tendon, and confirming it radiologically during the operation.

Our procedure primarily rests upon the adequacy of the reduction achieved before we start the procedure. This prevents the excessive use of image intensifier. In cases where closed reduction was not achieved, we did not hesitate in performing an open reduction with a mini incision. The distal locking was done by a free-hand technique. The reason of malunion in intramedullary nailing was failure to centralize the nail in wide distal tibial metaphysis. Considering the utility of poller screws as proposed by Krettek *et al*.,^19^ we used poller screw in seven cases in which the alignment and stability were not satisfactory. In unstable fractures of the lower tibia with associated fibular fracture, we resorted to plating the fibular fracture and locking the nail within 15 mm of the lower tibial articular surface. This has helped in solving the problems associated with unstable fractures. We agree that fibular plate fixation increased the initial rotational stability after distal tibial fracture compared with that provided by tibial intramedullary nailing alone.^20^

Based on our results and experience with 148 cases and comparing the results with those published in the literature, we conclude that the results [Table 4] were excellent in 90 cases (60.8%), good in 50 cases (33.8%), and fair in eight cases (5.4%). We strongly advise that the modified Kuntscher nail provides an effective implant providing stability and allowing union in simple and complex tibial fractures (it is our observation based upon the analysis of the patients in our series). Toe touch weight-bearing for a period of 6 weeks allows early osseous healing without fatigue, and failure of locking bolts^21^ was not seen in our study. Fear of shortening because of dynamization and early weight-bearing has not been sustained. The mechanically incompetent and biologically viable fragments heal around the nail to promote union and early recovery.

**Table 4:** The comparative, published studies of different authors

Authors	R/UR	n	Union week	Infection (%)	NU (%)	MU (%)	Joint stiffness	Compartment syndrome
Brown *et al.* (1990)^2^	R	125	17	1.6	1.6	2.4	7.2	1.6
Djahangiri (2006)^5^	R	119	24	2.1	9.4	9.4	0	6.3
Gregory *et al.* (1995)^4^	UR	47	24	2.6	8	0	0	0
Our current study	UR	148	17	3.3	2.7	8	0	1.3

R: reamed, UR: Unreamed, Nu=Nonunion, Mu=Malunion

Our system refers to a dynamic osteosynthesis because only distal locking is done after adequate reduction, to prevent the rotational malalignment and allow stability. Dynamic locking refers to the trans fixation only in the shorter fragment and allowing intermittent compression of the fracture site during early weight-bearing. The D nail provides sufficient stability by three-point fixation at either ends and also in the diaphysis by its bony contact. We believe that to achieve uneventful union with minimum morbidity, due attention should be focused on avoiding damage to the periosteum of small bone fragments in comminuted fractures.^22^ It has been proved^19^ that minimal surgical trauma and flexible fixation allow prompt healing when the blood supply to bone is maintained or can be restored early.

Our aim was to describe a cost-effective (implant costing less than US$10), simple method of dynamic osteosynthesis with the provision of interlocking, which we hope shall find a place in the majority of armamentarium used in the management of the fractures of the tibial diaphysis. A short period of external splintage (patellar tendon bearing cast) does not jeopardize the ultimate result.

This is not a comparative study between any other fixation methods. It seems plausible to say that D nail seems to be a good technique of fixation, with quick learning curve, a low-cost implant adhering to the principles of biological osteosynthesis.
